# The Importance of Cincho-Quinine as a Remedy

**Published:** 1877-09

**Authors:** 


The Importance of Cinco-Quinine as a Remedy.—The Supervising General of the Marine Hospital Service has issued a circular letter to the medical officers of that branch of the Treasury in which he calls their attention to the extraordinary increase in the market price of sulphate of quinia, and at the same times alludes to the success attending the employment of the other alkaloids of the bark.

In the year 1866 the Madras Government appointed a Medical Commission to test the respective efficacy in the treatment of fevers of quinine, quinidine, cinchonine, and the remedial value of these four alkaloids as deduced from their experiments is shown by the following statement:

| | | |
|---|---|---|
| Quinidine | ratio of failure per 1,000 cases | 6 |
| Cinchonidine, | " " " " " " | 10 |
| Quinine, | " " " " " " | 7 |
| Cinchonine, | " " " " " " | 23 |

Cincho-quinine contains all these alkaloids, and the combination has proved more efficacious than any one alone; and the price of this article being less than one-half the present price of the sulphate of quinine, the physicians of this country are substituting it for the sulphate; and the medical officers of the Government service should give this subject due consideration in preparing their requisitions for medical supplies.—*Washington, D. C., Daily Nation, August* 8, 1877.

